# Antiviral Activity of Type I, II, and III Interferons Counterbalances ACE2 Inducibility and Restricts SARS-CoV-2

**DOI:** 10.1128/mBio.01928-20

**Published:** 2020-09-10

**Authors:** Idoia Busnadiego, Sonja Fernbach, Marie O. Pohl, Umut Karakus, Michael Huber, Alexandra Trkola, Silke Stertz, Benjamin G. Hale

**Affiliations:** a Institute of Medical Virology, University of Zurich, Zurich, Switzerland; b Life Science Zurich Graduate School, ETH and University of Zurich, Zurich, Switzerland; Icahn School of Medicine at Mount Sinai

**Keywords:** ACE2, COVID-19, SARS-CoV-2, coronavirus, immunotherapy, interferons, receptors

## Abstract

Severe acute respiratory syndrome coronavirus 2 (SARS-CoV-2), the cause of coronavirus disease 2019 (COVID-19), is a recently emerged respiratory coronavirus that has infected >23 million people worldwide with >800,000 deaths. Few COVID-19 therapeutics are available, and the basis for severe infections is poorly understood. Here, we investigated properties of type I (β), II (γ), and III (λ1) interferons (IFNs), potent immune cytokines that are normally produced during infection and that upregulate IFN-stimulated gene (ISG) effectors to limit virus replication. IFNs are already in clinical trials to treat COVID-19. However, recent studies highlight the potential for IFNs to enhance expression of host angiotensin-converting enzyme 2 (ACE2), suggesting that IFN therapy or natural coinfections could exacerbate COVID-19 by upregulating this critical virus entry receptor. Using a cell line model, we found that beta interferon (IFN-β) strongly upregulated expression of canonical antiviral ISGs, as well as ACE2 at the mRNA and cell surface protein levels. Strikingly, IFN-λ1 upregulated antiviral ISGs, but ACE2 mRNA was only marginally elevated and did not lead to detectably increased ACE2 protein at the cell surface. IFN-γ induced the weakest ISG response but clearly enhanced surface expression of ACE2. Importantly, all IFN types inhibited SARS-CoV-2 replication in a dose-dependent manner, and IFN-β and IFN-λ1 exhibited potent antiviral activity in primary human bronchial epithelial cells. Our data imply that type-specific mechanisms or kinetics shape IFN-enhanced ACE2 transcript and cell surface levels but that the antiviral action of IFNs against SARS-CoV-2 counterbalances any proviral effects of ACE2 induction. These insights should aid in evaluating the benefits of specific IFNs, particularly IFN-λ, as repurposed therapeutics.

## Abstract (precis)

Repurposing existing, clinically approved, antiviral drugs as COVID-19 therapeutics is a rapid way to help combat the SARS-CoV-2 pandemic. Interferons (IFNs) usually form part of the body’s natural innate immune defenses against viruses, and they have been used with partial success to treat previous new viral threats, such as HIV, hepatitis C virus, and Ebola virus. Nevertheless, IFNs can have undesirable side effects, and recent reports indicate that IFNs upregulate the expression of host ACE2 (a critical entry receptor for SARS-CoV-2), raising the possibility that IFN treatments could exacerbate COVID-19. Here, we studied the antiviral- and ACE2-inducing properties of different IFN types in both a human lung cell line model and primary human bronchial epithelial cells. We observed differences between IFNs with respect to their induction of antiviral genes and abilities to enhance the cell surface expression of ACE2. Nevertheless, all the IFNs limited SARS-CoV-2 replication, suggesting that their antiviral actions can counterbalance increased ACE2.

## OBSERVATION

Severe acute respiratory syndrome coronavirus 2 (SARS-CoV-2) is the etiological agent of coronavirus disease 2019 (COVID-19), a new infectious respiratory disease characterized by a broad range of symptoms categorized as mild (fever, cough, mild pneumonia), severe (labored breathing [dyspnea], respiratory distress, and pneumonia), or critical (respiratory failure, septic shock, and multiple organ failure) (1, 2). Since its presumed emergence in late 2019, pandemic SARS-CoV-2 has caused over 23 million laboratory-confirmed human infections worldwide, and at least 800,000 deaths (3), predominantly in those over 70 years of age or with preexisting comorbidities, such as cardiovascular disease, diabetes, chronic respiratory disease, hypertension, or cancer (2). There are currently no specific licensed antiviral treatments or proven vaccines available for COVID-19, and the molecular basis for many severe SARS-CoV-2 infections is poorly understood.

Angiotensin-converting enzyme 2 (ACE2) is the essential receptor for SARS-CoV-2 entry into host cells (4, 5), and it is therefore a critical determinant of viral replication and pathogenesis. ACE2 protein is not expressed by all human cells, but it has been found in high abundance on the surfaces of type II alveolar epithelial cells (AT2 pneumocytes) in the lower parts of the human respiratory tract (6) and at low levels on ciliated airway epithelial cells of the nose and trachea (7). Recent single-cell sequence analysis efforts have sought to systematically identify specific human cell subsets that express *ACE2* mRNA (8–10), with the aim of determining the precise cellular tropism of SARS-CoV-2 and linking this with pathology. Intriguingly, *ACE2* mRNA levels also appear to be regulated by a multitude of factors, including age (11), tobacco smoke (12), and other respiratory tract infections (e.g., rhinovirus and influenza virus) (9) potentially via the canonically antiviral type I and II interferon (IFN) pathways (9, 12, 13). Thus, a tempting hypothesis that remains to be experimentally validated is that external or biological factors leading to enhanced *ACE2* expression may exacerbate SARS-CoV-2 replication and/or expand its cellular tropism (14), thereby increasing the severity of COVID-19 in individuals of certain risk groups, including the elderly, smokers, and those with coinfections or comorbidities (15).

## 

### Type I and III IFNs restrict SARS-CoV-2 replication in primary human bronchial epithelial cells.

**FIG 1:**
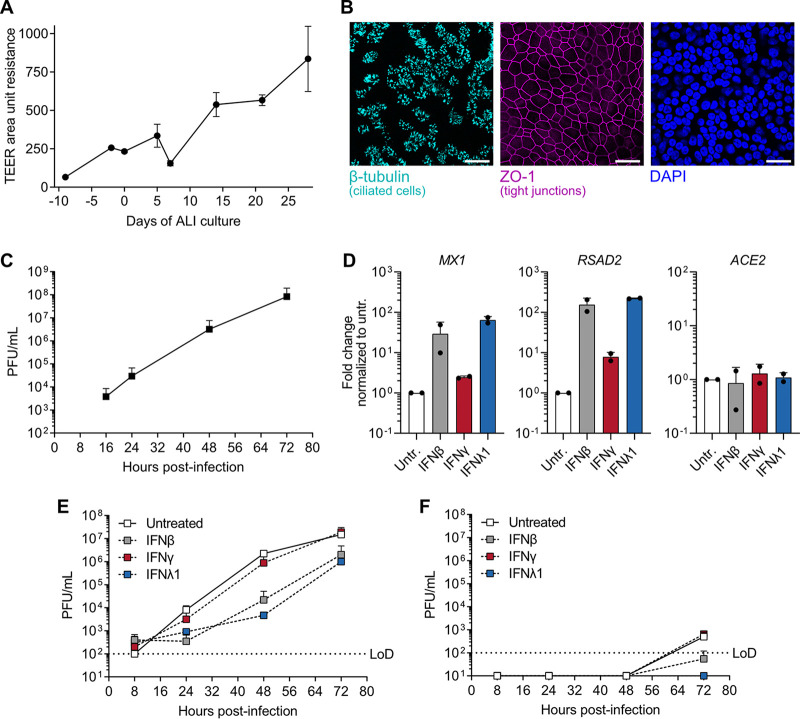
Replication of SARS-CoV-2 in primary human differentiated bronchial epithelial cells and its restriction by type I, II, and III interferons. (A) Primary human bronchial epithelial cells (BEpCs) were differentiated and grown at an air-liquid interface (ALI) for 28 days. TEER was monitored at the time points indicated, with data representing means ± standard deviations (error bars) from three independent wells. (B) Differentiated BEpCs were fixed and stained for ciliated cells (β-tubulin; turquoise), tight junctions (ZO-1; magenta) and nuclei (4′,6′-diamidino-2-phenylindole [DAPI]; blue). Bars, 25 μm. (C) Differentiated BEpCs grown on 6.5-mm filter inserts were infected with 6,000 PFU of SARS-CoV-2 from the apical side. At the indicated times postinfection, apical washes were harvested and virus titers were determined by plaque assay. Data represent means plus standard deviations from two independent replicates. (D) Differentiated BEpCs were treated on the apical side with 1,000 IU/ml type I (β), type II (γ), or type III (λ1) IFN (or left untreated [Untr.]) for 16 h before total RNA was harvested and the levels of *MX1*, *RSAD2* and *ACE2* were quantified by RT-qPCR. Data represent means plus standard deviations from two independent replicates. (E and F) Differentiated BEpCs were treated with 1,000 IU/ml type I (β), type II (γ), or type III (λ1) IFN (or left untreated) for 16 h prior to infection with 6,000 PFU of SARS-CoV-2 from the apical side. At the indicated times postinfection, apical washes (E) and basolateral samples (F) were harvested and virus titers were determined by plaque assay. Data represent means plus standard deviations from two independent replicates. The limit of detection (LoD) is indicated by the dotted lines.

Given that many respiratory viruses cocirculating with SARS-CoV-2 (such as influenza virus, rhinovirus, or seasonal coronaviruses) can trigger host IFN production, as well as the recent repurposing of IFNs in clinical trials as COVID-19 therapeutics (16), we sought to understand the interplay between the antiviral action of IFNs (17), the IFN-stimulated expression of ACE2 (9, 12, 13), and SARS-CoV-2 replication. As ACE2 expression was reported to be predominantly induced by type I and II IFNs in primary human cells (9), we first established a primary human bronchial epithelial cell (BEpC) SARS-CoV-2 infection model. BEpCs were grown at an air-liquid interface (ALI) and validated for their differentiation into a pseudostratified respiratory epithelium by measuring increased transepithelial electrical resistance (TEER) and presence of epithelium-specific cell and tight junction markers, such as β-tubulin and zona occludens protein 1 (ZO-1) (Fig. 1A and B). We found that these differentiated human BEpCs were highly permissive to SARS-CoV-2 replication when infected from the apical side, mimicking the natural route of initial infection, and viral titers reached in excess of 10^7^ PFU/ml at 72 h postinfection (Fig. 1C). The BEpCs could readily mount an antiviral gene expression profile (e.g., increased *MX1* and *RSAD2*) following 16 h stimulation with type I (β), II (γ), or III (λ1) IFNs, but surprisingly, we did not observe induction of *ACE2* with any of the IFNs tested, in contrast to other recent reports (9, 12, 13) (Fig. 1D). We note that our BEpC donor was a 73-year-old “never-smoker” and that age can correlate with ACE2 levels (11), presumably via an IFN-independent mechanism, leading us to hypothesize that the lack of *ACE2* induction by IFNs in our BEpC system could be due to relatively high basal levels saturating potential expression changes. Nevertheless, pretreatment of BEpCs with type I and III, but not type II, IFNs for 16 h lead to a clear reduction in SARS-CoV-2 replication following apical side infection (Fig. 1E). Furthermore, at 72 h postinfection, a small amount of infectious SARS-CoV-2 was detected on the basolateral side of the BEpC cultures in the absence of treatment, but this was blocked by type I and III IFNs (Fig. 1F). These data indicate that type I and III IFNs exert potent IFN-stimulated gene (ISG) induction and antiviral activity against SARS-CoV-2 in primary human bronchial epithelial cells.

### IFN-induced ACE2 is not sufficient to enhance SARS-CoV-2 replication in the presence of IFN-induced antiviral activity.

**FIG 2:**
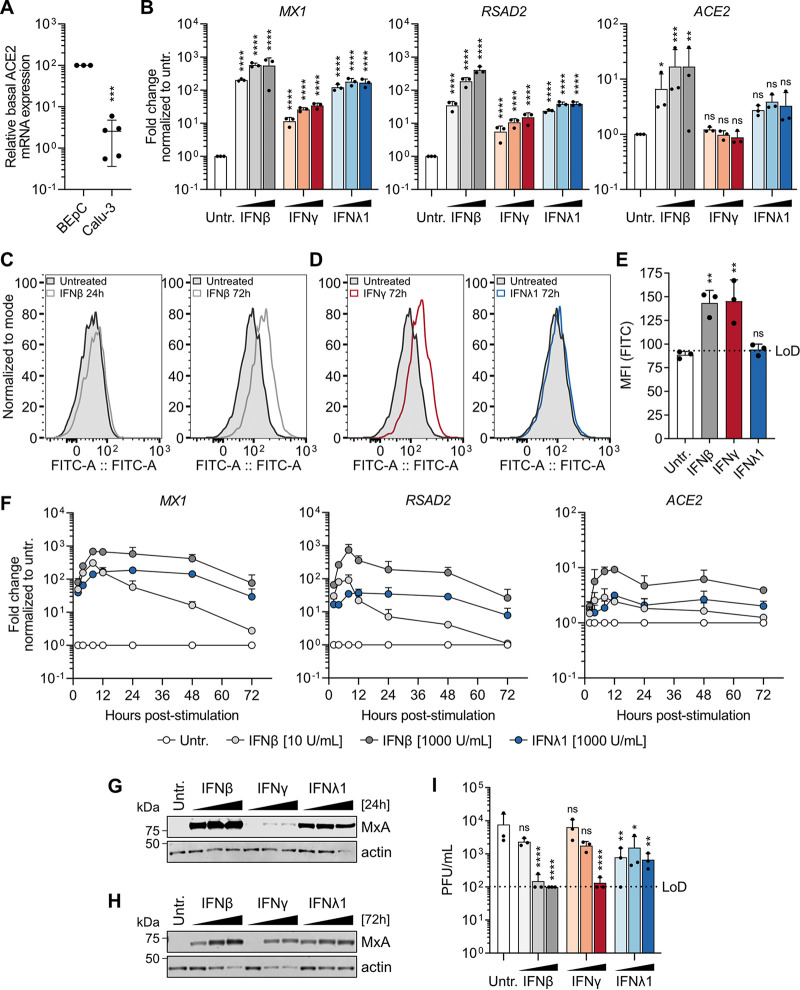
Antiviral activity of type I, II, and III interferons counterbalances ACE2 induction to restrict SARS-CoV-2 replication in Calu-3 cells. (A) Basal *ACE2* mRNA expression in BEpCs and Calu-3 cells was measured by RT-qPCR, and the levels in BEpCs were set at 100% for comparison. Data represent means ± standard deviations from three independent BEpC samples and five independent Calu-3 samples. (B) Calu-3 cells were treated with 10, 100, or 1,000 IU/ml of type I (β), type II (γ), or type III (λ1) IFN (or left untreated [Untr.]) for 16 h before total RNA was harvested and mRNA levels of *MX1*, *RSAD2*, and *ACE2* were determined by RT-qPCR. Data represent means plus standard deviations from three independent replicates. Statistical significance was determined for cells treated with each IFN compared to untreated cells using two-way ANOVA following log transformation (ns, not significant; *, *P* < 0.05; **, *P* < 0.002; ***, *P* < 0.0002; ****, *P* < 0.0001). (C) Calu-3 cells were treated with 1,000 IU/ml of IFN-β for 24 h (left) or 72 h (right) before ACE2 surface levels were assessed by FACS. (D) Calu-3 cells were treated with 1,000 IU/ml of IFN-γ (left) or IFN-λ1 (right) for 72 h before ACE2 surface levels were assessed by FACS. (E) Mean fluorescence intensity (MFI) values from Calu-3 cells treated with 1,000 IU/ml of type I (β), type II (γ), or type III (λ1) IFN (or left untreated [Untr.]) for 72 h and analyzed by FACS for ACE2 surface levels. Data represent means plus standard deviations from three independent replicates. The limit of detection (LoD) is indicated by the dotted line. Statistical significance was determined for cells treated with each IFN compared to untreated cells by one-way ANOVA (ns, not significant; **, *P* < 0.0002). (F) Calu-3 cells were treated with the indicated amounts of type I (β) or type III (λ1) IFN (or left untreated [Untr.]) before total RNA was harvested at the indicated times and mRNA levels of *MX1*, *RSAD2*, and *ACE2* were determined by RT-qPCR. Data represent means plus standard deviations from three independent replicates. (G and H) Calu-3 cells treated for 24 h (G) or 72 h (H) with 10, 100, or 1,000 IU/ml of type I (β), type II (γ), or type III (λ1) IFN (or left untreated [Untr.]) were lysed, and the levels of MxA and actin were determined by Western blotting. Data shown are representative of two independent replicates. (I) Calu-3 cells treated for 72 h with 10, 100, or 1,000 IU/ml of type I (β), type II (γ), or type III (λ1) IFN (or left untreated [Untr.]) were infected with SARS-CoV-2 at an MOI of 1 PFU/cell. Virus was harvested at 24 h postinfection, and titers were determined by plaque assay. Data represent means plus standard deviations from three independent replicates. Statistical significance was determined for cells treated with each IFN compared to untreated cells by two-way ANOVA following log transformation (ns, not significant; *, *P* < 0.05; **, *P* < 0.002; ****, *P* < 0.0001).

We tested the hypothesis that lack of *ACE2* induction by IFNs in the primary BEpC cultures was due to relatively high basal levels saturating potential changes. Indeed, by comparative real-time quantitative PCR (RT-qPCR) analysis of *ACE2* in primary BEpCs and the human lung epithelial cell line model Calu-3, we noted that basal expression of *ACE2* at the bulk population level was substantially higher in the primary BEpCs than in Calu-3 cells (Calu-3s) (Fig. 2A). Furthermore, stimulation of Calu-3s with type I IFN for 16 h led to a dose-dependent induction of *ACE2* gene expression, increasing *ACE2* mRNA levels approximately 20-fold (Fig. 2B). Calu-3 cells were also found to be susceptible to SARS-CoV-2 infection (see Fig. S1A in the supplemental material), and consistent with a previous report (18), SARS-CoV-2 infection triggered blunted type I/III IFN responses in these cells as defined by levels of *IFNB1*, *IFNL1*, *MX1*, and *RSAD2* (Fig. S1B to E). Consistent with this, SARS-CoV-2 infection alone was also unable to induce *ACE2* mRNA levels, unlike type I IFN (Fig. S1F). We therefore used Calu-3s as an amenable cell line model with IFN-inducible *ACE2* to dissect the interplay between IFNs, ACE2, and SARS-CoV-2 further.

10.1128/mBio.01928-20.1 FIG S1 Infection of Calu-3 cells with SARS-CoV-2 results in blunted type I and III interferon responses. Calu-3 cells were infected (or mock infected) with SARS-CoV-2 at an MOI of 5 PFU/cell or were treated (or left untreated [untr.]) with 1,000 IU/ml of type I (α2) interferon (IFN). At the indicated times postinfection or posttreatment, total RNA was harvested and mRNA levels of SARS-CoV-2 *E* (A), human *IFNB1* (B), human *IFNL1* (C), human *MX1*(D), human *RSAD2* (E), and human *ACE2* (F), were determined by RT-qPCR. Data represent means and standard deviations from three independent replicates. Download FIG S1, TIF file, 0.6 MB. Copyright © 2020 Busnadiego et al. 2020 Busnadiego et al. This content is distributed under the terms of the Creative Commons Attribution 4.0 International license.

While type I, II, and III IFNs all induced canonical ISGs (such as *MX1* and *RSAD2*) in Calu-3s, there was a gradient to this response, with type I IFN being a more potent or rapid inducer than type III IFN and type II IFN inducing the lowest levels of ISGs. A similar pattern was noted for IFN-induced *ACE2*, although *ACE2* induction at the mRNA level was not detectable for type II IFN, and it was slightly increased for type III IFN albeit this did not reach statistical significance (Fig. 2B). To understand whether *ACE2* mRNA induction translated into increased ACE2 protein levels on the surfaces of cells, we analyzed IFN-treated Calu-3 cells by fluorescence-activated cell sorting (FACS). While type I IFN potently induced *ACE2* mRNA at 16 h posttreatment, we could detect increased cell surface expression of ACE2 protein only after treatment of cells for 72 h, and not for earlier time points (Fig. 2C). In line with the low induction of *ACE2* mRNA by type III IFN, we were unable to detect ACE2 cell surface expression following treatment with this IFN, even after 72 h treatment (Fig. 2D and E). Surprisingly, type II IFN, which did not lead to detectable induction of the *ACE2* gene (Fig. 2B), clearly increased cell surface expression levels of ACE2 protein in Calu-3s after 72 h treatment (Fig. 2D and E), suggesting either different kinetics for *ACE2* mRNA induction by this IFN or a transcription-independent mechanism for protein upregulation. Indeed, kinetic and dose-response analyses of a wide range of canonical ISGs and *ACE2* after type I and III IFN treatments revealed that type I IFN induced faster and greater gene expression responses than type III IFN, though following comparable gene induction type III responses appeared to be of longer duration (Fig. 2F and Fig. S2A to C). Importantly, expression of the canonical antiviral ISG marker protein, MxA, was maintained throughout the prolonged IFN treatment time necessary to detect enhanced surface expression of ACE2 (Fig. 2G and H). We therefore pretreated Calu-3s for 72 h with each of the IFNs in a dose-dependent manner with the aim of inducing different levels of ACE2 cell surface expression, as well as the canonical antiviral ISG program, prior to infection with SARS-CoV-2. Strikingly, treatment with type I, II, and III IFNs all inhibited the replication of SARS-CoV-2 in Calu-3s, despite the previously observed enhanced ACE2 cell surface expression caused by type I and II IFNs (Fig. 2I). Notably, SARS-CoV-2 replication in the Calu-3 cell model system in the absence of IFN was relatively low (reaching only ∼10^4^ PFU/ml at 24 h postinfection), suggesting that an increase in virus replication caused by enhanced ACE2 expression would have been detected in our set-up. These data indicate that, while IFNs can upregulate ACE2 at both the gene expression and cell surface levels, the antiviral action of IFNs is more powerful and counterbalances ACE2 induction to restrict SARS-CoV-2 replication.

10.1128/mBio.01928-20.2 FIG S2 Dose and kinetic interferon-stimulated gene response of Calu-3 cells following type I and III interferon treatment. Calu-3 cells were treated with the indicated amounts of type I (β) or type III (λ1) IFN (or left untreated [Untr.]) before total RNA was harvested at the indicated times and mRNA levels of *DDX58* (A), *IFIT1* (B), and *IFIT2* (C), were determined by RT-qPCR. Data represent means and standard deviations from three independent replicates. Download FIG S2, TIF file, 0.3 MB. Copyright © 2020 Busnadiego et al. 2020 Busnadiego et al. This content is distributed under the terms of the Creative Commons Attribution 4.0 International license.

### Concluding remarks.

Due to the lack of antiviral drugs that specifically target the newly emerged SARS-CoV-2, rapid repurposing of existing licensed drugs with other modes of action is a critical tool to combat the pandemic. IFNs are pleotropic cytokines that exhibit broad-spectrum antiviral activity through the upregulation of hundreds of ISGs; therefore, IFN therapy could be a treatment option for COVID-19 patients. To date, type I IFNs (IFNα-2b, IFNβ-1a, and IFNβ-1b) and type III IFN (IFN-λ1a) have all begun clinical trials as potential COVID-19 therapeutics (16, 19, 20). However, several recent studies have indicated that IFNs upregulate the expression of ACE2 (9, 12, 13), the critical SARS-CoV-2 receptor (4), suggesting that IFN treatments could have the potential to exacerbate virus replication under some circumstances. The data presented here confirm that IFNs can induce *ACE2* mRNA expression and further suggest type-specific differences between IFNs with respect to their promotion of ACE2 protein expression at the cell surface, the site relevant for infection. The molecular basis for these differences between IFNs, or whether differences simply reflect the speed, magnitude, and duration of individual responses, remains to be dissected. Nevertheless, in both the Calu-3 cell line model and primary human bronchial epithelial cells, IFNs exhibited inhibitory activity against SARS-CoV-2, indicating that the antiviral function of IFNs counterbalances any possible replication-promoting benefit of increased ACE2 expression. While these findings cannot necessarily be extrapolated to all biological tissue types or other *in vivo* conditions, together with similar recent studies (21–23), they do support the concept that selected IFNs could be used as short-term antiviral therapies until new specific, potent COVID-19 drugs are developed (16). Notably, IFN-λ, which has critical antiviral functions in the respiratory and gastrointestinal tracts (22, 24) and which appears to exhibit less detrimental proinflammatory effects compared to other IFNs (25), did not induce a detectable increase in ACE2 protein cell surface expression in our hands, despite sustained canonical antiviral ISG induction. This may add weight to proposals that IFN-λ is a superior therapeutic candidate against SARS-CoV-2 than other IFNs.

### Experimental procedures.

**(i) Cell lines and interferons.** Calu-3 and Vero-CCL81 cells (ATCC) were cultured at 37°C and 5% CO_2_ in Dulbecco’s modified Eagle’s medium (DMEM; Gibco), supplemented with 10% (vol/vol) fetal calf serum (FCS), 100 U/ml of penicillin, and 100 μg/ml of streptomycin (catalog no. 15140-122; Gibco). Recombinant IFN-α2 (catalog no. NBP2-34971; Novusbio), IFNβ-1b (catalog no. NBP2-35892; Novusbio), IFN-γ (catalog no. NBP2-34992; Novusbio), and IFN-λ1 (catalog no. 300-02L; Peprotech) were used at the indicated concentrations. For pretreatment of primary human bronchial epithelial cells (BEpCs), IFNs were added to the apical compartment in phosphate-buffered saline (PBS). All incubations were performed at 37°C.

**(ii) Differentiation of primary human bronchial epithelial cultures.** Primary human bronchial epithelial cells from a 73-year-old female donor were purchased from Promocell (catalog no. C-12640). Cells were grown in airway epithelium basal growth medium (catalog no. C-21260l; Promocell) supplemented with an airway growth medium supplement pack (catalog no. C-39160; Promocell) and 10 μM Y-27632 (Selleck Chemicals). For differentiation, transwell plates with 6.5-mm polyester filter inserts (catalog no. CLS3470; Corning) were coated with collagen: a 0.5-mg/ml collagen (catalog no. C7774; Sigma-Aldrich) stock in 0.5 M acetic acid (catalog no. 100063.1000; Merck) was diluted to 0.15 mg/ml in PBS prior to coating the filters. BEpCs were seeded onto the coated transwell filters in a 1:1 mixture of airway epithelium basal growth medium and DMEM, which was supplemented with an airway growth medium supplement pack, and cells were grown until confluence was reached. For differentiation at the air-liquid interface (ALI), medium was removed from the apical compartment, and the medium in the basal compartment was supplemented with 150 ng/ml retinoic acid (catalog no. R2625; Sigma-Aldrich). BEpCs were cultured at ALI for a minimum of 28 days prior to use, and medium in the basal compartment was refreshed every 3 days.

**(iii) Validation of differentiated primary human bronchial epithelial cultures.** Differentiation of the airway cultures was verified by regularly measuring the transepithelial electrical resistance (TEER) using an ERS-2 meter (Millicell). In addition, the presence of ciliated cells and tight junctions was verified by immunofluorescence. Briefly, cells were fixed with 3.7% paraformaldehyde in PBS and permeabilized with PBS supplemented with 50 mM ammonium chloride (catalog no. 254134; Sigma-Aldrich), 0.1% saponin (catalog no. 47036; Sigma-Aldrich) and 2% bovine serum albumin (BSA) (catalog no. A7906; Sigma-Aldrich). A mouse anti-β-tubulin IV antibody (ab11315; Abcam) and a rabbit anti-ZO-1 antibody (catalog no. 61-7300; Thermo Fisher Scientific) were used to stain ciliated cells and tight junctions, respectively. As secondary antibodies, anti-mouse IgG Alexa Fluor 488 and anti-rabbit IgG Alexa Fluor 546 antibodies (catalog no. A-21202 and catalog no. A-11035; Thermo Fisher Scientific) were used. Filters were mounted using ProLong Gold Antifade Mountant (catalog no. P36930; Thermo Fisher Scientific), and images were acquired on an SP5 confocal microscope (Leica).

**(iv) SARS-CoV-2 isolation and stocks.** SARS-CoV-2 (strain IMV5 [SARS-CoV-2/human/Switzerland/IMV5/2020]) was isolated on Vero-CCL81 cells from an anonymized patient nasopharyngeal swab sample that had tested PCR positive during routine diagnostics at the Institute of Medical Virology, University of Zurich in March 2020. Briefly, 100 μl of a fivefold dilution series of sample in serum-free DMEM was mixed with 1 × 10^5^ Vero-CCL81 cells in 500 μl of DMEM supplemented with 10% FCS, 100 U/ml penicillin, 100 μg/ml streptomycin, and 2.5 μg/ml amphotericin B (catalog no. 15290018; Gibco). Cells were seeded in 24-well plates and incubated at 37°C for 4 to 5 days until cytopathic effect was apparent. Supernatants were centrifuged at 1,500 rpm for 5 min, and 250 μl of this cleared supernatant (termed passage 1 [P1]) was used to inoculate a 25-cm^2^ flask of freshly seeded Vero-CCL81 cells, which were then cultured in the same way for a further 4 or 5 days at 37°C. Cell supernatants were harvested, clarified by centrifugation at 1,500 rpm for 5 min, and aliquoted before freezing at −80°C (termed P2). P2 stocks were verified by our in-house diagnostics service to be PCR positive for SARS-CoV-2. Following titer determination by plaque assay (see below), a P3 working stock was generated by infecting Vero-CCL81 cells at a multiplicity of infection (MOI) of 0.001 PFU/cell for 72 h in DMEM supplemented with 100 U/ml penicillin, 100 μg/ml streptomycin, 0.3% bovine serum albumin (BSA) (catalog no. A7906; Sigma-Aldrich), 20 mM HEPES (catalog no. H7523; Sigma-Aldrich), 0.1% FCS, and 0.5 μg/ml tosylsulfonyl phenylalanyl chloromethyl ketone (TPCK)-treated trypsin (catalog no. T1426; Sigma-Aldrich), prior to supernatant clarification by centrifugation (1,500 rpm, 5 min), aliquoting/storage at −80°C, and plaque titration. All work with infectious SARS-CoV-2 was performed in an approved biosafety level 3 (BSL3) facility by trained personnel at the Institute of Medical Virology, University of Zurich. All procedures and protective measures were thoroughly risk assessed prior to starting the project and were approved by the Swiss Federal Office of Public Health (Ecogen number A202808/3).

**(v) SARS-CoV-2 plaque assays.** To determine SARS-CoV-2 titers by plaque assay, Vero-CCL81 cells in 12-well plates were infected with 100 μl of 10-fold serial dilutions of sample in PBS supplemented with 0.3% BSA, 1 mM Ca^2+^/Mg^2+^, 100 U/ml penicillin, and 100 μg/ml streptomycin. After 1 h at 37°C with regular rocking, inoculum was removed and replaced with 1 ml/well of agar overlay (MEM; 0.1% sodium bicarbonate, 0.01% DEAE dextran, 0.6% oxoid agar, and 1 mg/ml TPCK-trypsin). Once the overlay had solidified, plates were incubated at 37°C for 72 h prior to fixing with 3.7% formaldehyde in PBS. Cell monolayers were stained with crystal violet (0.5% crystal violet and 20% methanol) to visualize plaques.

**(vi) SARS-CoV-2 infection assays.** For Calu-3 experiments, 5 × 10^4^ cells were seeded overnight in 24-well plates before treatment with the indicated IFN. Cells were then infected with SARS-CoV-2 at the indicated MOI in PBS supplemented with 0.3% BSA, 1 mM Ca^2+^/Mg^2+^, 100 U/ml penicillin, and 100 μg/ml streptomycin. After 1 h inoculation, cells were washed once in PBS, and the medium was replaced with DMEM supplemented with 100 U/ml penicillin, 100 μg/ml streptomycin, 0.3% BSA, 20 mM HEPES, 0.1% FCS, and 0.5 μg/ml TPCK-treated trypsin. Samples were taken at the indicated time points, and supernatants were stored at −80˚C prior to titer determination. For BEpC experiments, cells were infected from the apical side with 6,000 PFU SARS-CoV-2 in PBS supplemented with 0.3% BSA, 1 mM Ca^2+^/Mg^2+^, 100 U/ml penicillin, and 100 μg/ml streptomycin. After 1 h, the inoculum was removed, and cells were incubated at 37°C. At selected time points, virus was harvested from the apical side by applying 80 μl of PBS for 15 min at 37°C before removal and storage at −80°C prior to titer determination. Basolateral samples were also collected and frozen at −80˚C.

**(vii) Assessment of RNA levels by RT-qPCR.** Total RNA was extracted from cells using the ReliaPrep RNA cell miniprep system (Promega) according to the manufacturer’s instructions. cDNA was synthesized from 1 μg of total RNA using Superscript III reverse transcriptase (Thermo Fisher) and oligo(dT)_15_ primer (Promega). RT-qPCR was performed on a 7300 real-time PCR system (Applied Biosystems) using Fast EvaGreen qPCR master mix kit (Biotium) and the following primers: for *MX1*, Hs.PT.58.26787898 (Integrated DNA Technologies); for *RSAD2*, 5′-CCCCAACCAGCGTCAACTAT-3′ and 5′-TGATCTTCTCCATACCAGCTTCC-3′; for *ACE2*, 5′-GGCTTGGGAAAGCTGGAGAT-3′ and 5′-GGGATGGCAGACTGCTTTCT-3′; for *IFNB1*, 5′-CATTACCTGAAGGCCAAGGA-3′ and 5′-CAGCATCTGCTGGTTGAAGA-3′; for *IFNL1*, 5′-GGTGACTTTGGTGCTAGGCT-3′ and 5′-TGAGTGACTCTTCCAAGGCG-3′; for *DDX58*, 5′-TGCAAGCTGTGTGCTTCTCT-3′ and 5′-TCCTGAAAAACTTCTGGGGCT-3′; for *IFIT1*, 5′-CTGTGGTAGGCTCTGCTTCC-3′ and 5′-CCACCACACCCAGCTAAGTT-3′; for *IFIT2*, 5′-GCGTGAAGAAGGTGAAGAGG-3′ and 5′-GCAGGTAGGCATTGTTTG-3′. The delta delta- cycle threshold (ΔΔCt) was determined relative to untreated or mock-infected samples. Gene expression was normalized to glyceraldehyde-3-phosphate dehydrogenase (GAPDH) (5′-CTGGCGTCTTCACCACCATGG-3′ and 5′-CATCACGCCACAGTTTCCCGG-3′). qPCR for the SARS-CoV-2 *E* gene was performed by our in-house diagnostics service using the TaqMan method (26) and the following primers: 5′-ACAGGTACGTTAATAGTTAATAGCGT-3′ (forward [fwd]), 5′-ATATTGCAGCAGTACGCACACA-3′ (reverse [rev]), and 5′-ACACTAGCCATCCTTACTGCGCTTCG-3′ (probe).

**(viii) Assessment of ACE2 cell surface levels by flow cytometry.** Following the appropriate treatment, ∼2 × 10^5^ Calu-3 cells were detached from 24-well plates with 0.25% trypsin-EDTA (Thermo Fisher Scientific) and resuspended in DMEM supplemented with 10% FCS and penicillin-streptomycin. Cells were washed twice with FACS buffer (PBS supplemented with 1 mM EDTA [Thermo Fisher Scientific] and 2% BSA [Sigma-Aldrich]) by centrifugation at 1,100 rpm for 3 min at 4°C and resuspension of cell pellets. To stain for surface ACE2, cells were incubated in 50 μl FACS buffer containing 4 μg/ml of anti-human ACE2 antibody (catalog no. AF933-SP; R&D Systems) and 10 μg/ml rabbit anti-goat IgG (H+L) cross-adsorbed secondary antibody, DyLight 488 (catalog no. SA5-10078; Thermo Fisher Scientific), for 1 h at 4°C. To exclude dead cells, stain from a LIVE/DEAD Fixable Near-IR Dead Cell Stain kit (Thermo Fisher Scientific) was used at a dilution of 1:1,000. Cells were further washed three times with FACS buffer, resuspended in 200 μl of FACS buffer, and analyzed on a FACSVerse system (BD). Typically, 3 × 10^3^ to 5 × 10^3^ live cells were acquired, and fluorescein isothiocyanate (FITC) intensities were examined.

**(ix) SDS-PAGE and Western blotting.** Cell lysates were prepared in 2× urea disruption buffer (6 M urea, 4% sodium dodecyl sulfate [SDS], 1 M β-mercaptoethanol, bromophenol blue), sonicated, and heated to 95°C for 5 min. Proteins were separated by SDS-polyacrylamide gel electrophoresis (PAGE) on 4 to 12% NuPAGE Bis-Tris gradient gels (Life Technologies) and transferred to nitrocellulose membranes (GE Healthcare, Amersham). The indicated proteins were detected using antibodies specific for MxA (mouse ab143; kindly provided by Jovan Pavlovic), or actin (rabbit) (catalog no. A2103; Sigma-Aldrich). The secondary antibodies used were IRDye 800CW goat anti-mouse IgG (catalog no. 926-32210; Li-Cor) and IRDye 680RD goat anti-rabbit IgG (catalog no. 926-68071; Li-Cor). A Li-Cor Odyssey scanner was used for detection.

**(x) Statistical analyses.** Statistical analyses were performed using GraphPad Prism 8.4 (GraphPad Software, San Diego, CA, USA). Data were log transformed and analyzed by either one-way or two-way analysis of variance (ANOVA) for multiple comparisons. *P* values for significance are given in the figure legends.
