# Supplementary figures and images for: Antiviral Activity of Type I, II, and III Interferons Counterbalances ACE2 Inducibility and Restricts SARS-CoV-2
Source: mBio. 2020 Sep 10;11(5):e01928-20. doi: 10.1128/mBio.01928-20 (PMC7484541; doi:10.1128/mBio.01928-20)

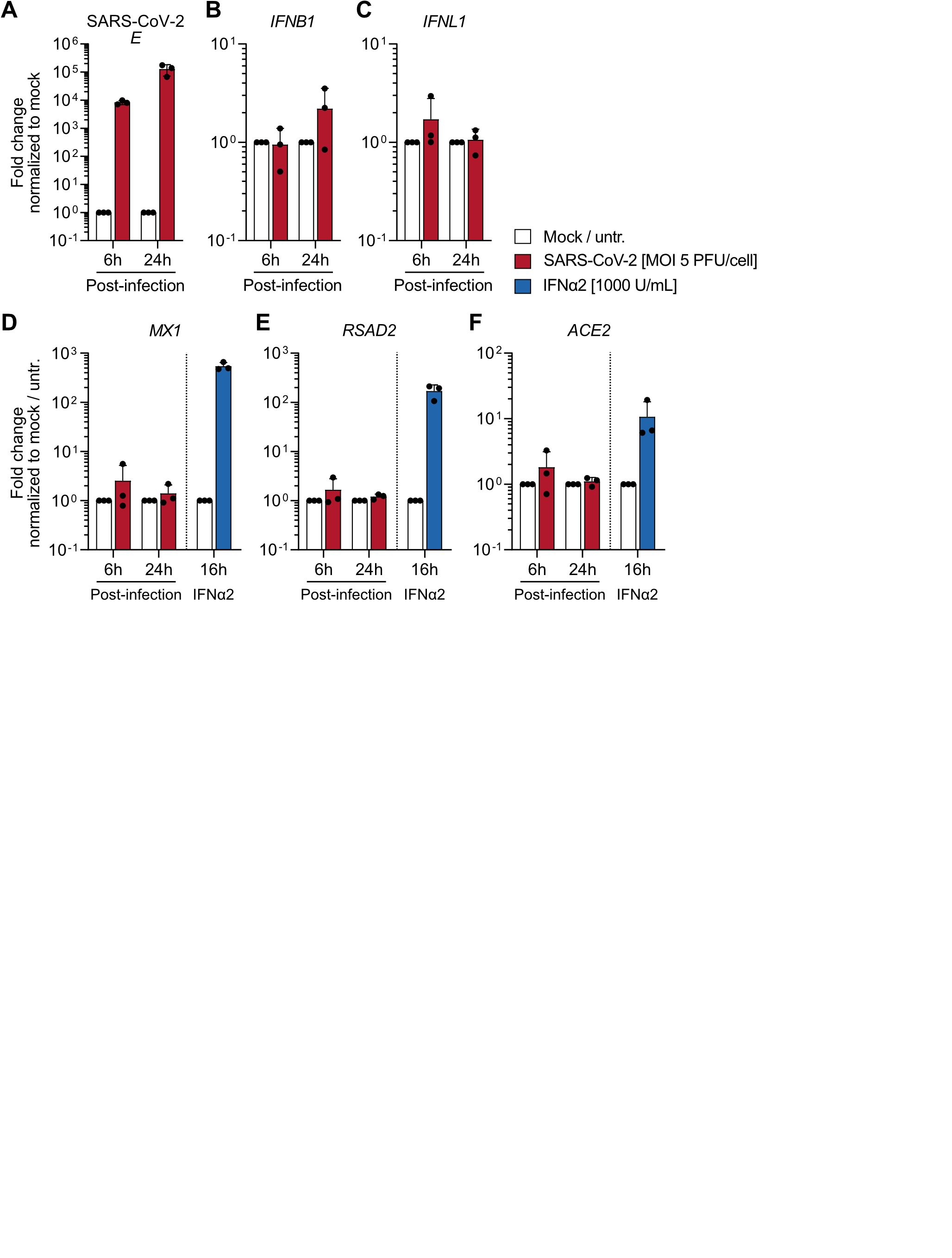

Supplement: FIG S1 [file mBio.01928-20-sf001.tif]

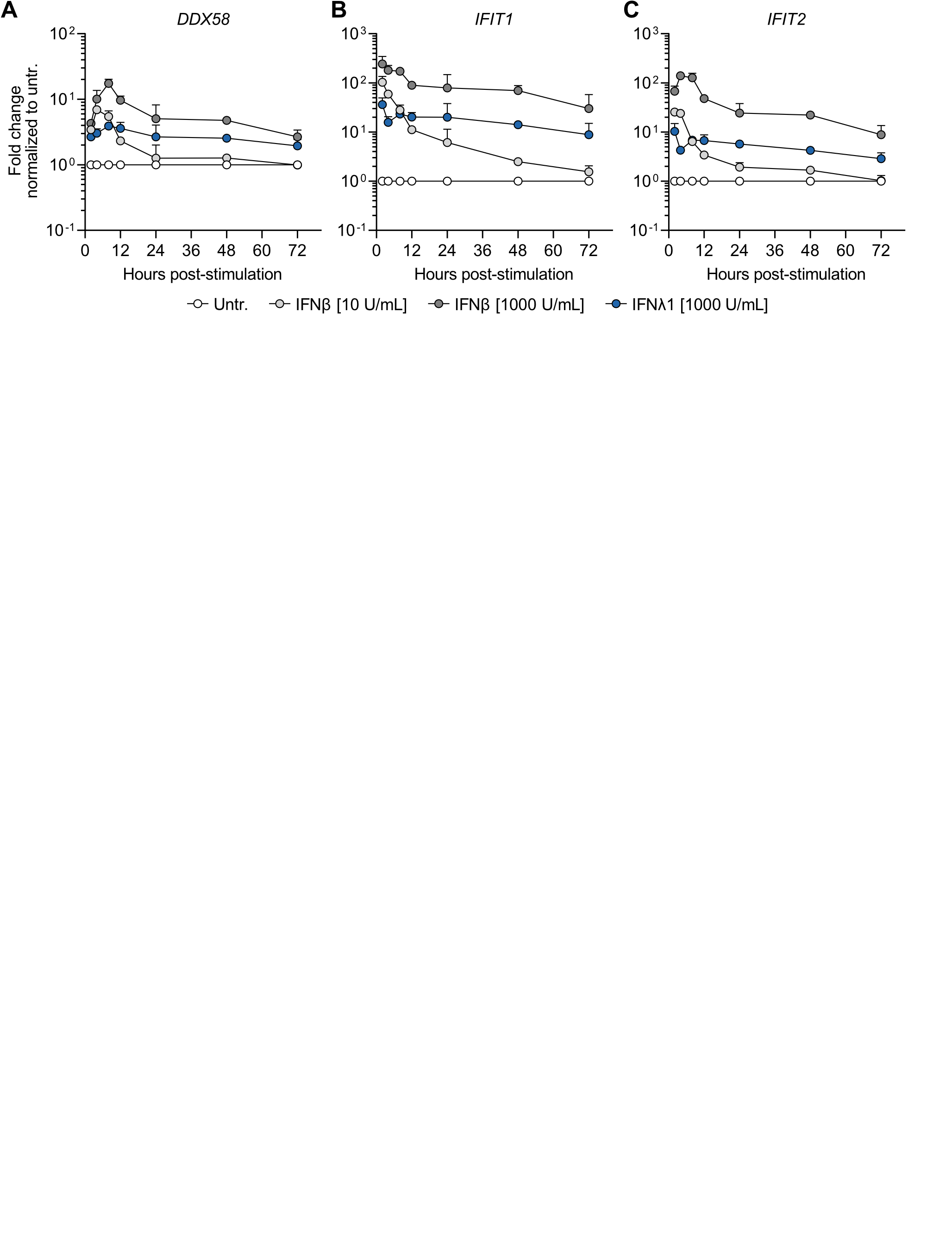

Supplement: FIG S2 [file mBio.01928-20-sf002.tif]
